# A structured tool for delivering feedback on medical student clinical clerkings

**DOI:** 10.15694/mep.2019.000200.1

**Published:** 2019-11-12

**Authors:** Nikesh Devani, Vasileios Gkiousias, Oliver Mitchell, Joseph Walton, Tereze Bogdanova, Efthimia Karra, Nick Murch, Paul Dilworth

**Affiliations:** 1 Royal Free London NHS Foundation Trust; 2 University College London Medical School

**Keywords:** Feeback, Clinical Clerkings, Undergraduate Medical Education, Structured Appraisal Tool

## Abstract

This article was migrated. The article was marked as recommended.

**Introduction:** The initial history and examination is a fundamental aspect of clinical practice. Most medical students cultivate this skill through regular undertaking of ‘clerkings’ during their clinical placements. We designed a written, structured, proforma-based approach to delivery of feedback on student clerkings which also promoted the undertaking of a ‘complete clerking’ encouraging students to maintain a whole-system holistic approach. Within this paper, we present our findings following its introduction at a London teaching hospital.

**Methods:** Sixty-one medical students on their first clinical attachment within acute medicine were asked to submit at least one full medical clerking for objective appraisal using the structured clerking feedback proforma by a clinical teaching fellow. Students completed a ‘pre’ and ‘post’ assessment using Likert Scales at the time of receiving their clerking feedback. Structured interviews of randomly selected students and senior medical educators were also undertaken.

**Results:** Following introduction of the structured feedback proforma, there was a significant increase across all indices of student-perceived utility and satisfaction compared to previously received feedback (which was mostly ad-hoc verbal). Using Likert Scales (1 to 10: 1 representing least effect and 10 representing greatest effect) student assessment of usefulness was 9.0 (versus 6.34 for previous feedback); likelihood of influencing future practice was 8.8 (versus 6.47); extent to which it reinforced the message of a complete clerking was 9.5 (versus 6.13) and extent to which the feedback would encourage them to undertake complete clerkings was 9.0. Free text comments and subsequent interviews of randomly selected students and senior medical educators reinforced the positive perception of this approach.

**Conclusions:** The introduction of a structured clerking feedback proforma can improve the quality and utility of the feedback delivered to medical students on their acute medical clerkings and can promote and reinforce the value of maintaining a whole-system holistic approach.

## Introduction

The initial history and examination is a fundamental aspect of clinical practice ( Hampton *et al.*, 1975; Maguire and Rutter, 1976). It is therefore essential that doctors possess the skills needed to extract, interpret and document information from patients to effectively manage their presenting problems. This skill is refined throughout a clinician’s career but first taught when at medical school. Most medical schools provide formal training to students before clinical encounters centred around how to undertake a thorough history and clinical examination (the ‘clerking’) and require students to complete a minimum number of these to demonstrate competency. However, despite their importance, students often only receive appraisal on their written clerkings in an ad-hoc informal basis or verbally during educational meetings. This approach does not always provide an optimal environment for learning and development.

It is well recognised that feedback occupies a catalytic role in the learning process ( Boehler *et al.*, 2006) yet most studies continue to demonstrate that students believe it to be poorly executed and given less attention by clinical educators ( Ende, 1983; Maggs, 2012). This reflects the discord in the knowledge and expectations of what constitutes effective feedback between educators and students ( Mulliner and Tucker, 2015). However, when performed well, feedback evokes both affective (e.g. promoting motivation and engagement) and cognitive (e.g. alleviating cognitive burden and allowing for reconfiguration of existing information and beliefs) responses ( Hattie and Timperley, 2007; Shute, 2008). Via the aid of feedback, students can be directed towards expected goals, good practice can be reinforced and areas for further development can be highlighted ( Ende, 1983). Studies have shown that students appreciate the value and usefulness of feedback that is simple, clear, task-driven and delivered in a timely manner and by a credible source ( Perera *et al.*, 2008; Watling *et al.*, 2012). Furthermore, in their RCT, Boehler *et al* (2006) identified that specific, constructive feedback improved medical student performance beyond that of generic, compliment based feedback ( Boehler *et al.*, 2006). Feedback is also of vital importance to educators as it provides an insight into their teaching effectiveness and signposts the requirement for any adjustments to better meet the needs of students ( Urquhart, Ker and Rees, 2018).

To harness the clear utility of feedback and address current shortcomings in its provision, particularly in the context of appraising medical student clinical clerkings, we developed an innovative approach to its delivery. We devised a structured feedback proforma (Supplementary File 1) which appraised a student clerking across various domains in a simple, clear and standardised manner. The proforma was arranged in the same order as a traditional clerking (e.g. presenting complaint, history of presenting complaint, past medical history etc.) and provided the educator with a framework for appraisal thereby ensuring consistency in approach. It allowed the educator to communicate areas of good practice and encouraged them to provide meaningful, constructive and specific suggestions for development. Furthermore, the proforma promoted the undertaking of a ‘complete clerking’ whereby students were actively encouraged to maintain a holistic, whole-systems approach throughout rather than being siloed into one system based on the patient’s presenting complaint. Within this paper, we describe the outcomes following introduction of the proforma at one clinical placement site within a London Medical School.

## Method

The London Medical School provides early clinical years education at three main teaching-hospital campuses. Students in their fourth academic year who were embarking on their first clinical attachment at ‘Campus R’ were subjected to the novel clerking feedback proforma. During their placement on the acute medical unit, these students were requested to submit at least one full medical clerking for formal appraisal using the new proforma. The completion of the proforma and delivery of feedback was undertaken by a medical clinical teaching fellow. Upon receipt of this feedback, students were asked to complete a ‘pre’ and ‘post’ questionnaire to assess their satisfaction and the perceived utility of this and any previously received feedback. The questionnaires utilised numerical Likert scales ranging from 1 to 10, with 1 representing least effect and 10 representing greatest effect across a range of domains (listed in Table 1).

**Table 1.** Components of pre- and post-questionnaires used to gather student feedback

‘Pre’ Questionnaire
How useful has any previous feedback on clinical clerkings been?
In what format has previous feedback on clinical clerkings been delivered? (e.g. verbal, written..)
How influential was the feedback you received on your approach to subsequent clerkings you performed?
To what extent has feedback you’ve previously received reinforce the message of a complete clerking *?
**‘Post’ Questionnaire**
Overall, how useful is the feedback you have just received on your clinical clerking?
How clearly has the feedback been presented and structured?
How likely do you feel that the feedback you have received will change the way you approach futureclerkings?
To what extent does the structured feedback you have just received reinforce the message of a complete clerking *?
To what extent will this feedback encourage you to undertake ‘complete’ clerkings’ in the future?

* ‘Complete clerkings’ defined within questionnaires as: *‘a thorough full systems history and clinical examination, generation of a problems list and differential diagnoses with a comprehensive investigation and management plan addressing all of the problems identified.’*

Five further cohorts of students rotating through acute medicine at Campus R were also studied. Following collation of all student-feedback data, statistical analysis using the a Mann-Witney U test for non-parametric variables was applied to determine significance in the differences observed between the ‘pre’ and ‘post’ assessments of feedback utility and satisfaction. Structured interviews of randomly selected students and senior medical educators were undertaken in parallel. Furthermore, a survey of students across the other 2 campuses (not exposed to the new feedback proforma) was also performed to determine appetite for such an intervention.

## Results

In total, 61 medical students received appraisal on their medical clerking using the new structured feedback proforma. Prior to the introduction of this intervention, only 63% of these students report having received any feedback on their clerkings and this was mostly an informal verbal appraisal (87%). An assessment of utility and relevance of this prior feedback revealed scores of 6.3 for usefulness; 6.4 for likelihood that the feedback will influence future practice; and 6.1 for extent to which feedback reinforced the message of a complete clerking. ( Table 2).

Following receipt of the completed structured feedback proforma, students rated the usefulness of this structured feedback as 9.0; the likelihood of it influencing future practice was rated 8.9; the extent to which it reinforced the message of a complete clerking as 9.5 and the extent to which the feedback would encourage them to undertake complete clerkings as 9.0. A summary of this data has been provided in Table 2. When compared, the structured feedback proforma approach produced statistically significant differences across Likert measures of usefulness, influence and reinforcement of key learning message (all P values < 0.0001).

**Table 2.** Comparison of Student Satisfaction with feedback received before and after implementation of the structured clerking feedback proforma

Descriptor	Satisfaction with feedback received prior to proforma (‘pre’). Mean (SD)	Satisfaction with structured proforma feedback (‘post’). Mean (SD)	P-value
	*Likert Scales 1 - 10 (with 1 representing least effect and 10 greatest effect)*		
**Usefulness of feedback**	6.34 (1.6)	9.0 (1.1)	<0.0001
**Clarity of feedback presentation and structure**	n/a	9.4 (1.0)	
**Likelihood feedback will influence approach to future clerkings**	6.47 (1.7)	8.8 (1.3)	<0.0001
**Extent to which feedback reinforced message of a ‘complete clerking’**	6.13 (1.9)	9.5 (0.9)	<0.0001
**Extent to which feedback will encourage undertaking of ‘complete clerkings’ in the future**	n/a	9.0 (1.2)	

Free text comments and subsequent structured interviews of randomly selected students (n=3) reinforced the positive perception of this approach. Of those interviewed, Student 1 commented that the *‘detailed and structured feedback received acted as an excellent guide for future clerkings and.. felt more genuine, thoughtful and credible than much of the feedback previously received.’* Student 2 ‘ *appreciated the emphasis that the proforma placed on providing supportive, useful and constructive advice. and that they used the feedback to help address uncertainties they had at the start of their first clinical placement.’* Student 3 reflected that ‘ *the proforma probably acted as a useful tool for the doctor providing the feedback: it served as a form of a training for them highlighting what should constitute feedback on clerkings and what is useful to know for students.’*

A survey of those students (n=10) based at other placement sites not yet using the structured feedback proforma revealed that 90% would have liked to receive it in this manner. These students expressed concerns regarding ‘uncertainty about the structure and constitution of an ideal clerking,’ ‘a lack of preparedness for future clinical practice’ and ‘difficulty formulating a differential and effective management plan’ as some of the shortcomings associated with a general verbal feedback approach which they had received.

Structured interviews of 2 senior medical educators (Clinician 1 and 2) provided a faculty perspective on the structured clerking feedback proforma. Clinician 1 commented that the *‘the* *proforma introduced a consistent approach in assessing students. By minimising inter-rater variability and confusion as to what the assessment criteria are, it acted as a platform for standardisation and harmonisation of practice.’*

Clinician 2 reflected on the utility of the proforma as a learning tool for students: *‘the proforma promotes the concept of adopting a holistic approach when clerking patients and allows appreciation of which elements of clerking should be focussed on.’*

Clinician 1 and 2both remarked on the practical application of the proforma: *‘The proforma is an easy-to-use tool and allows for formative assessment at the end of clinical rotations. It can allow identification of those students that perform well and those who might need more support as well as highlighting specific areas that require improvement if the latter is the case.’*

## Discussion

Our study demonstrates that a structured, written approach to delivering feedback on acute medical clerkings results in higher student satisfaction than verbal or unstructured written feedback which was previously delivered. The feedback is perceived as more useful and of higher quality and is more likely to promote the important message of maintaining a whole-system holistic approach throughout a clerking. The structured feedback proforma achieves these attributes through generation of feedback which is practically usable, detailed and ‘personalised to the student’s own work’ - attributes which are recognised as desirable in Dawson *et al’s* (2019) large scale survey of Australian University Students ( Dawson *et al.*, 2019).

Our feedback proforma is intuitive and easy to use for both educators and learners; it requires minimal training and can be readily adapted and implemented to complement a medical school’s existing curriculum. The proforma might also be tailored for different specialty requirements, for example surgical specialities; children or women’s health where the focus and composition of a clerking might differ slightly. Furthermore, it is well established that feedback providers must possess the appropriate knowledge and skills to present feedback in an effective manner ( Prins, Sluijsmans and Kirschner, 2006). Through its clear yet comprehensive structure, our proforma has the advantage of also acting as a learning aid for feedback providers signposting them towards what ideally should be included in order to deliver effective and useful feedback to students.

The structured proforma allows a standardised and transparent approach to delivery of feedback and can therefore help temporise inter-educator variability and the discord in the perceptions and expectations of feedback between educators and learners ( Mulliner and Tucker, 2015). In the context of acute medical clerkings, this ensures consistent reinforcement of best practice methods, and validity and equity in assessment for all learners. Student-perceived equity might stem from the understanding that completion of the proforma requires the educator to identify areas for improvement. The student might therefore be more likely to expect and accept constructive comments them rather than perceive them as unfair criticism. The latter might not be true of informally delivered verbal criticism - certainly in the study by Boehler *et al.* (2006), students appeared to be more ‘satisfied’ with general compliments than with ‘feedback on deficiencies ( Boehler *et al.*, 2006).’ Use of a standardised proforma which signposts towards the receipt of constructive criticism might make this more acceptable and hence useful for students.

A limitation of this study is the reliance on student satisfaction as a marker of feedback proforma effectiveness. However, this approach proved most practical because alternatives, such as measuring the proforma’s effect on student clinical performance, are difficult to determine conclusively and will be affected by a multitude of other factors. Perhaps this represents an area for further research. A further limitation is the involvement of students at only a single institution - we attempted to address this by also collecting opinions from students at other clinical placement sites and performing structured interviews of senior medical educators. In addition, we plan to further our research into the utility of this structured approach by introducing the proforma within other medical specialties and across different clinical year groups based at a variety of hospital sites.

In conclusion, the results of our study suggest that the introduction of a structured clerking feedback proforma can improve the quality and utility of the feedback delivered to medical students. It can promote the important message of performing a complete clerking and provide a clear framework to allow the student to develop this skill further. This will support the student working towards achieving many of the ‘professional skills’ requirements listed within the GMC’s recent ‘Outcomes for Graduates’ publication ( GMC, 2018). Furthermore, through the provision of constructive and personalised feedback encompassed in a learning culture, students will gradually develop a deeper understanding and appreciation of such feedback, ultimately leading to the creation of competent, confident and reflective clinicians.

## Take Home Messages

• The use of a structured tool to appraise medical student clinical clerkings results in the generation of constructive feedback which is practically usable and can guide future learning needs.
• Structured, proforma-based feedback is perceived as more useful and of higher quality by medical students and permits a standardised and transparent approach to feedback delivery.
• The structured tool is intiutive and can act as an aide-memoir for educators delivering feedback.
• A structured feedback proforma can encourage the adoption of a whole-system ‘holistic’ approach to medical student clerkings.

## Notes On Contributors

Dr Nikesh Devani is a Respiratory Registrar at the Royal Free London NHS Foundation Trust within the North East London Deanery and former Clinical Teaching Fellow for University College London Medical School.

Dr Vasileios Gkiousias is a Core Surgical Trainee and current University College London Clinical Teaching Fellow.

Dr Oliver Mitchell is a Foundation Year 2 Doctor within the North East London Deanery.

Dr Joseph Walton is an Acute Medical Registar at the Royal Free London NHS Foundation Trust within the North East London Deanery and a current Clinical Teaching Fellow for University College London Medical School.

Ms Tereze Bogdanova is a Senior Teaching Assistant and Module Manager at University College London Medical School.

Dr Efthimia Karra is a Consultant in Diabetes and Endocrinology & Acute Medicine at the Royal Free London NHS Foundation Trust and Undergraduate Royal Free Site Deputy Sub-Lead for University College London Medical School.

Dr Nick Murch is a Consultant and Clinical Lead in Acute Medicine at the Royal Free London NHS Foundation Trust, an Honorary Clinical Associate Professor at University College London Medical School and Acute Medicine Training Programme Director for NC/E London.

Dr Paul Dilworth is a Consultant Respiratory Physician at the Royal Free London NHS Foundation Trust and Undergraduate Royal Free Site Sub-Dean for University College London Medical School.
