# Supplementary material for: A structured tool for delivering feedback on medical student clinical clerkings
Source: MedEdPublish (2016). 2019 Nov 12;8:200. [Version 1] doi: 10.15694/mep.2019.000200.1 (PMC10712456; doi:10.15694/mep.2019.000200.1)
Supplement: Peer review history

# Reviewer report: Reviewer response for version 1

**DOI:** 10.21956/mep.19908.r30152

**Published:** 2020-06-28

**Authors:** Megan Anakin

**Affiliations:** 1 University of Otago

This review has been migrated. The reviewer awarded 3 stars out of 5 I read your article about the introduction and evaluation of the structured clerking feedback proforma with great interest. We are interested in improving the quality of feedback that students receive during their clinical placements at our medical school. The introduction provides a well-described background and rationale for the study. The pre-post questionnaire study design focused on student perceptions of the proforma was further enhanced with structured interviews with students and educators and an additional survey of students not involved with the proforma. The authors describe how the first source of data was analysed in the methods section but not the interview or additional survey data. The authors may wish to add this information to a revised version of their article. The authors may wish to enhance reporting the participant demographics for all three data sources since only the number of participants are given. Readers may appreciate information about gender, ethnicity, student age or experience prior to entry into the medical programme, and educator experience or discipline to help the reader contextualise the results and interpret the qualitative findings. For the results of the pre-post questionnaire data, the authors may wish to include an effect size so the readers can appreciate the magnitude of the difference between students pre- and post- proforma use ratings. Because the methods used to analyse the interview or additional survey data were not described, the reader cannot evaluate the representativeness and trustworthiness of the findings presented. For example, the reader cannot tell of the quotations presented from the survey data are all from one participant or from three different participants. The discussion focuses on the significance, implications, and applications of the acceptability by users of the proforma to enhance feedback. The inclusion of the proforma as a supplemental file will be useful for starting conversations with educators. Thank you for publishing this work.

Reviewer Expertise:

NA

No decision status is available

# Reviewer report: Reviewer response for version 1

**DOI:** 10.21956/mep.19908.r30154

**Published:** 2020-02-26

**Authors:** Ken Masters

**Affiliations:** 1 Sultan Qaboos University

This review has been migrated. The reviewer awarded 3 stars out of 5 An interesting paper discussing a structured tool for delivering feedback on medical student clinical clerkings. The authors begin by describing the importance of teaching history-taking, and the need for teaching it properly and then describe the use and evaluation of their feedback tool to assess the extent to which the students have mastered the process.The paper is very well and clearly presented, and the positive impact of the tool is reasonably clearly demonstrated through the statistical data. I would, however, like to have seen the qualitative data presented more formally, with themes identified from the comments, and then supported by data, rather than only a few tantalising comments taken from the small number of interviewed participants. One would expect that 61 students would be able to generate enough data for themes to be developed.Perhaps the authors would consider creating a Version 2 of their paper, in which they expand the qualitative data reporting.

Reviewer Expertise:

NA

No decision status is available

# Reviewer report: Reviewer response for version 1

**DOI:** 10.21956/mep.19908.r30150

**Published:** 2019-11-14

**Authors:** Sateesh Babu Arja

**Affiliations:** 1 Avalon University School of Medicine

This review has been migrated. The reviewer awarded 4 stars out of 5 I enjoyed reading this paper. The concept of structured feedback in clinical clerkship is a good phenomenon and need to be encouraged by every clinical educator. The literature review presented by the authors is up to date and relevant. However the concept of structured feedback in clerkship is a known phenomenon. Research methodology used by the authors in this study is appropriate. Data analysis and results are adequate. This manuscript is useful for all clinical educators.

Reviewer Expertise:

NA

No decision status is available

# Reviewer report: Reviewer response for version 1

**DOI:** 10.21956/mep.19908.r30151

**Published:** 2019-11-12

**Authors:** Carla Todaro

**Affiliations:** 1 Azienda Sanitaria Universitaria Integrata di Trieste

This review has been migrated. The reviewer awarded 5 stars out of 5 To be accepted a feedback has to be consistent, the use of a structured tool certainly represents a valid help for assessment and evaluation.On the other hand, the defined written evaluation criteria contribute to a better understanding by the students on their assessment and improve perception of equity during the evaluation process.This review underline the importance of a structured tools as help to deliver feedback and this can definitely be a valid help for who has not a previous experience on feedback delivering.

Reviewer Expertise:

NA

No decision status is available

# Reviewer report: Reviewer response for version 1

**DOI:** 10.21956/mep.19908.r30153

**Published:** 2019-11-12

**Authors:** Felix Silwimba

**Affiliations:** 1 University of Lusaka

This review has been migrated. The reviewer awarded 5 stars out of 5 this an educative study report. patient clerkship, history taking, physical examinations and investigations is very important to the competence of a trainee MD. I only wish a similar study can be applied in a non-native English speaking culture where English is the official language. I recommend this study to medical educators worldwide

Reviewer Expertise:

NA

No decision status is available
